# An activated‐zinc oral rinse reduces pro‐inflammatory cytokine secretion and promotes proliferation in *Porphyromonas gingivalis* LPS‐challenged gingival tissues – A pilot study

**DOI:** 10.1002/cre2.437

**Published:** 2021-05-06

**Authors:** Kaitlyn A. Stanton, Barbara A. McCracken

**Affiliations:** ^1^ Southern Illinois University School of Dental Medicine Alton Illinois USA

**Keywords:** immunohistochemistry, patient compliance, periodontitis

## Abstract

**Objectives:**

The use of adjunct post‐treatment mouth rinses containing chlorhexidine (CHX) for periodontitis patients is associated with side effects that reduce patient compliance. Our aim was to evaluate the proinflammatory and cell proliferation effects of an activated‐zinc mouth rinse (SM) that has been suggested as an alternative post‐treatment therapeutic.

**Materials and Methods:**

Tissue models of gingival epithelium were used to simulate periodontal disease and compare inflammatory reactions after treatment with CHX or SM. Tissues were exposed to *Porphyromonas gingivalis* LPS and wounded to simulate periodontal disease. Tissues were treated and incubated for 6, 12, or 24 h. Inflammatory cytokines were measured in culture medium by ELISA and local expression of Toll‐like receptor (TLR)‐4 and proliferation marker Ki‐67 was visualized by immunohistochemistry.

**Results:**

SM and CHX treatments decreased secretion of IL‐1β and IL‐8 into culture media at all time points. IL‐1β secretion levels were further decreased by SM compared to CHX treatment at all time points. TLR‐4 expression appeared significantly increased 12 h post‐treatment in the CHX tissues but remained relatively low in SM tissues at all time points. Ki‐67 results suggest that cell proliferation was increased in the SM tissues earlier than CHX tissues.

**Conclusions:**

Our data suggest that SM may reduce inflammation in gingival tissues.

## 1 INTRODUCTION

Periodontitis, an oral disease characterized by a polymicrobial infection and disproportional host response in the periodontal tissues, affects more than 47% of people worldwide and is the main cause of tooth loss (Eke et al., 2012). Without proper oral hygiene measures, a biofilm of microbial communities forms on tooth structures (Seneviratne et al., 2011). Inflammation is triggered in response to the increased biofilm, and changes to the microbial communities occur (Socransky & Haffajee, 2000a, 2000b). This reshaping of the microbiota from communities in homeostasis to dysbiosis may trigger further inflammation that ultimately results in periodontal disease (Lamont et al., 2018; Maekawa et al., 2014) However, recent re‐thinking of the relationships between specific bacteria and inflammation in periodontal disease suggests that it may be the inflammatory responses in susceptible individuals that drives the changes in the bacterial composition rather than the other way around (Bartold & Van Dyke, 2019). Either way, mechanical removal of the biofilm to encourage a reduction in inflammation remains the treatment of choice for periodontitis.

Mouth rinses containing chlorhexidine gluconate (CHX) have been shown to reduce biofilm development (Lang & Brecx, 1986). While some improvement in probing depths have been associated with CHX as an adjunct therapy for patients undergoing periodontal treatment, detrimental side effects may outweigh any benefit gained (da Costa et al., 2017). These side effects include brown staining of teeth and dental materials, changes in taste perception, and possible mucosal ulceration (Hepsø et al., 1988). SmartMouth Clinical DDS (SM) is an over‐the‐counter rinse that includes 0.05% cetylpyridinium chloride as an antimicrobial agent (Teng et al., 2016) and activated zinc to increase wound healing and control sulfur‐containing compounds associated with halitosis and periodontal disease. In a recent clinical comparison between SM and CHX in periodontitis patients prescribed a mouth rinse as an adjunct therapy during periodontal maintenance, patients assigned to SM showed better compliance. Furthermore, SM treatment was as effective as CHX in reducing gingival inflammation, bleeding, and plaque accumulation (Miley et al., 2019).

Therefore, we designed this in vitro study to test inflammatory and proliferation responses to SM and CHX in a tissue model of gingival epithelium. The hypothesis tested was that tissues in our periodontitis model would produce inflammatory cytokines and proliferate to repair damage in response to treatment with SM in a manner similar to or better than in response to treatment with CHX. The aim of the study was to provide support for the use of SM as an alternative to CHX in the management of patients requiring adjunct therapy.

## 2 METHODS

### 2.1 Tissue model of gingival epithelium

3D models of gingival mucosa (Epi‐Gin; GIN‐100, Epi‐Gingival™, MatTek Life Sciences, Ashland, MA) were obtained for use in this study. These tissue models are made from normal human oral keratinocytes derived from non‐diseased adults undergoing tooth extractions, or from cadavers. The three‐dimensional differentiated tissue is histologically similar to gingival mucosa and contains 8–11 layers of cells. To make the models, gingival cells are seeded into cell culture inserts (surface area of 0.6 cm; Seneviratne et al., 2011) that are coated with an extracellular matrix preparation. After several days of the tissue being submerged, the tissues are introduced to the air liquid interface to induce stratification and differentiation. At this point, tissue kits are shipped to the customer.

For this study, 2 kits of 24 tissues, for a total of 48 tissues, were used. Upon shipment arrival, tissues were placed in fresh serum‐free Dulbecco's Modified Eagle's Medium (DMEM) supplemented with epidermal growth factor, antibiotics, and a proprietary mix of growth factors (MatTek) overnight (37°C, 5% CO_2_) to rest and recover from shipping stress. Medium was placed under the culture inserts to permeate the porous membrane of the tissue culture inserts while the top layer remained at the air/liquid interface. The next day, one set of the tissues (24 tissues) was placed in DMEM media containing *Porphyromonas gingivalis* LPS (10 μg/ml; Millipore Sigma, St. Louis, MO), and the other in fresh DMEM without LPS for 24 h. A subset, 18 tissues, of each of sets was wounded with NaOH following a standardized protocol (MatTek; 0.5 μl 1 M NaOH for 15 min., https://www.mattek.com/wp-content/uploads/EpiCorneal-Wound-Healing-Application-Note.pdf). Epi‐Gin tissues, wounded (36) or non‐wounded (12), were then assigned to one of the 3 treatment groups. Each tissue was treated with 100 μl (standard product testing size, per MatTek) of SM, CHX, or phosphate buffered saline (PBS; control) for 30 s. The apical surface of the Epi‐Gin tissue was washed with 100 μl PBS to remove traces of mouth rinse. Tissues were incubated for 6, 12, or 24 h (2 tissues for each time point), fixed with 3% paraformaldehyde for 4 h at room temperature, and stored in PBS until processing. Tissue medium was collected for cytokine analysis at each time point and immediately frozen at ‐20°C until assays were conducted.

### 2.2 Enzyme‐linked immunosorbent assay

Human IL‐1β and Human IL‐8 ELISA kits (Invitrogen, Waltham, MA) were used to evaluate cytokine secretion following manufacturer's instructions. Briefly, 96‐well plates were coated with anti‐human IL‐1β or IL‐8 monoclonal antibodies. Serial dilutions of standards were included in each assay to obtain a standard curve. Samples were diluted according to manufacturer's suggestions and were added to the plates. After an overnight incubation, secondary antibodies were added following kit protocol. Absorbance at wavelengths of 450 nm and 570 nm were measured and concentrations calculated according to the standard curve. Concentration of IL‐1β and IL‐8 are expressed in pg/ml.

### 2.3 Immunohistochemistry staining

Fixed tissue samples were sent to MatTek for processing, paraffin embedding, and serial sectioning. Tissue sections were deparaffinized and rehydrated using decreasing concentrations of ethanol following standard protocol. TLR‐4 and Ki‐67 antibodies were added to separate tissue sections followed by incubation with biotinylated secondary antibodies (ABC Kit, Vector Laboratories, Burlingame, CA). DAB peroxidase substrate kit (Vector Laboratories) was used to visualize TLR‐4 and Ki‐67 tissue locations. Hematoxylin was used as a counterstain and glass coverslips were placed over the tissues.

## 3 RESULTS

Half of the 48 tissues were exposed to *P. gingivalis* LPS. Exposure to LPS was a significant driver of IL‐1β secretion in control tissues (only exposed to PBS; not wounded), but not of IL‐8 (Table 1). Concentrations of IL‐1β at 6, 12, and 24 h in tissues not exposed to LPS were 2.32, 0.69, and 0.28 pg/ml respectively. IL‐1β concentrations from tissues exposed to LPS, on the other hand, were 310.46, 290.84, and 135.72 pg/ml respectively (Table 1). Wounded tissues exposed to LPS showed a similar pattern with both IL‐1β and IL‐8 secretion (Table 1).

**TABLE 1:** Cytokine secretion (pg/ml ± SD) in non‐wounded and wounded tissues exposed to *Porphyromonas gingivalis* LPS (n = 2/group)

	6 h		12 h		24 h	
	+ LPS	− LPS	+ LPS	− LPS	+ LPS	− LPS
Not wounded						
IL1‐β	310.46 ± 20.6	2.32 ± 2.7	290.84 ± 24.9	0.69 ± 0.63	135.72 ± 0.04	0.28 ± 0.40
IL‐8	82.53 ± 57.2	96.31 ± 81.1	73.39 ± 10.0	53.06 ± 1.8	207.19 ± 0.0	85.75 ± 7.1
Wounded						
IL‐1β	134.19 ± 12.6	ND	60.23 ± 0.66	2.48 ± 1.3	43.79 ± 1.4	0.56 ± 0.79
IL‐8	112.06 ± 1.4	53.07 ± 0.68	106.41 ± 52.9	74.91 ± 30.4	383.65 ± 105.1	142.10 ± 13.5

**FIGURE 1:**
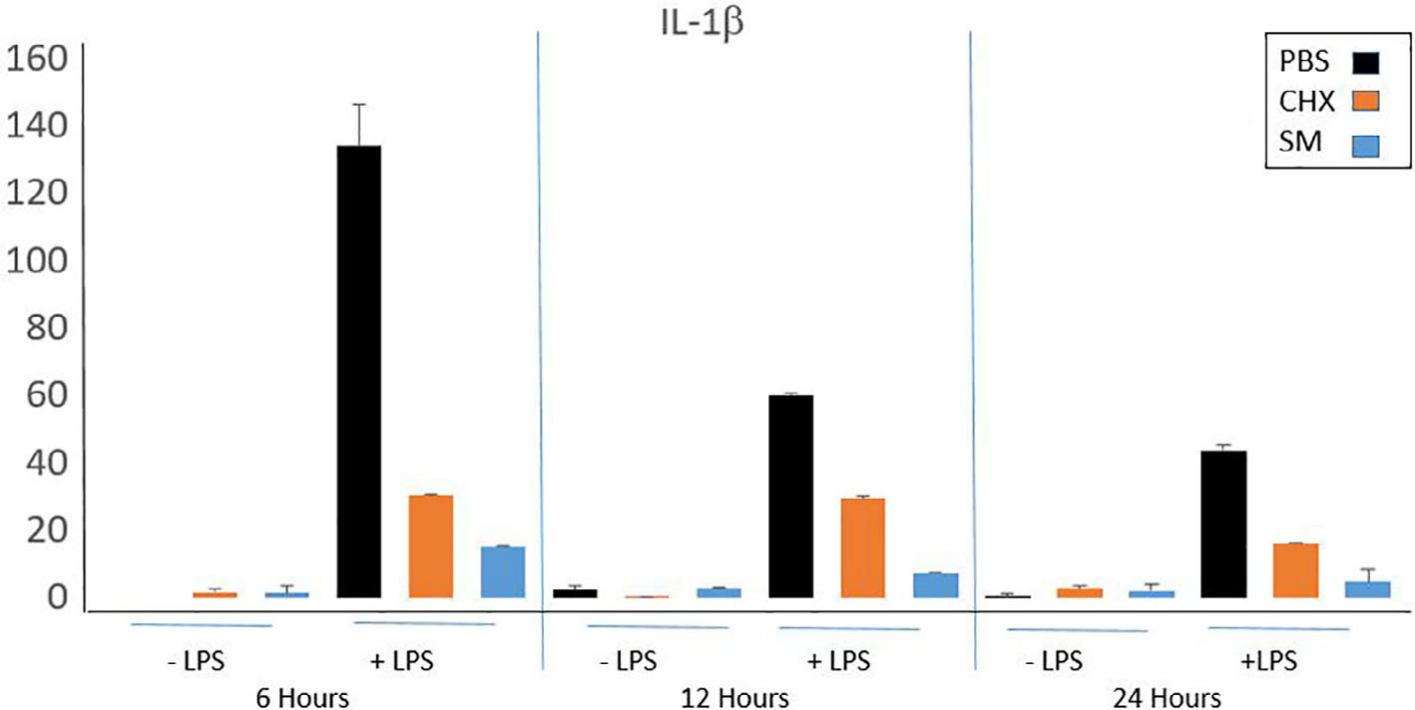
ELISA secretion of IL‐1β from wounded LPS‐challenged Epi‐Gingival tissues. IL‐1β secretion levels at 6, 12, and 24 h after treatment with respective mouth rinse. Error bars are standard deviation, *N* = 2

**FIGURE 2:**
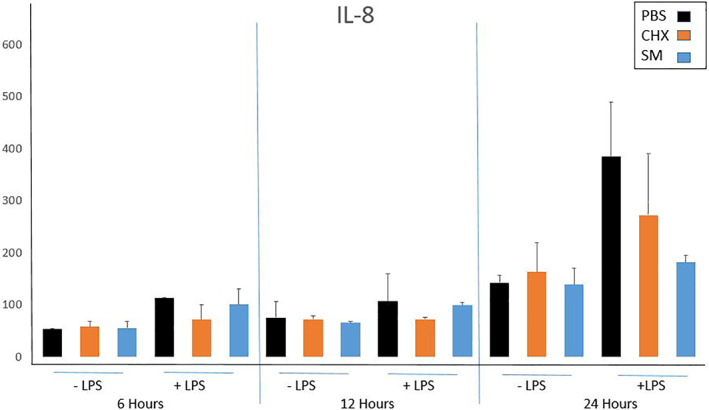
ELISA secretion of IL‐8 from wounded LPS‐challenged Epi‐Gingival tissues. Secretion of IL‐8 at 6, 12, and 24 h after treatment with respective mouth rinse. Error bars are standard deviations, *N* = 2

Both SM and CHX treatments showed a lower level of secretion of IL‐1β into tissue culture media at 6, 12, and 24 h time points compared to treatment with PBS in wounded tissues challenged with LPS (Figure 1). IL‐1β secretion levels were further decreased by SM compared to CHX treatment at all 3 timepoints (Figure 1). IL‐8 secretion generally increased after mouth rinse treatment (Figure 2).

**FIGURE 3:**
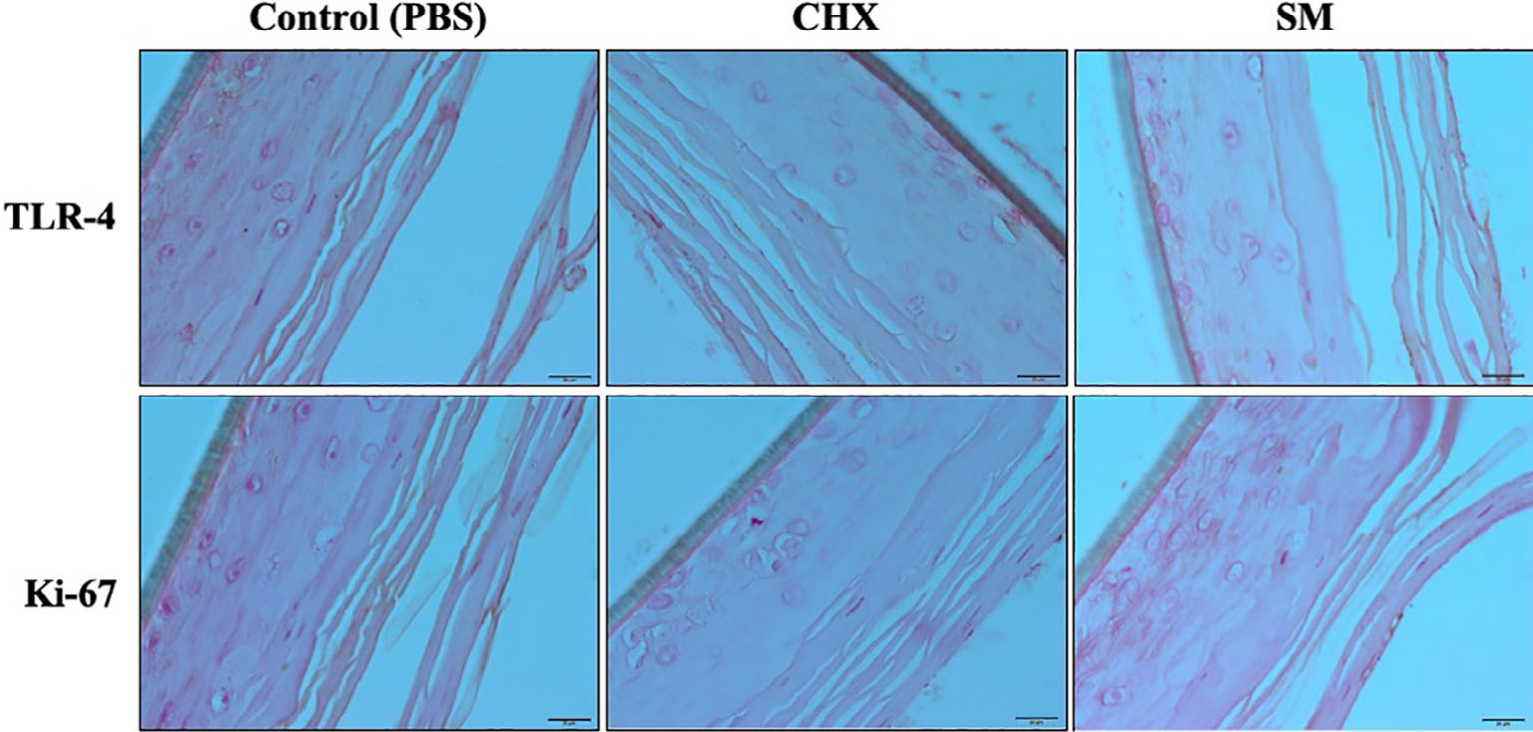
Immunohistochemistry images of wounded, LPS‐challenged Epi‐Gingival tissues showing TLR‐4 and Ki‐67 expression 6 h after respective treatments. Expression for TLR‐4 and Ki‐67 is represented by brown staining

**FIGURE 4:**
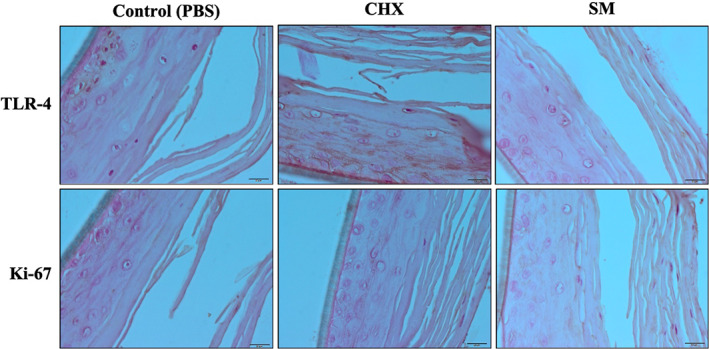
Immunohistochemistry images of LPS‐challenged Epi‐Gingival tissues showing TLR‐4 and Ki‐67 expression 12 h after respective treatment. Expression for TLR‐4 and Ki‐67 is represented by brown staining

**FIGURE 5:**
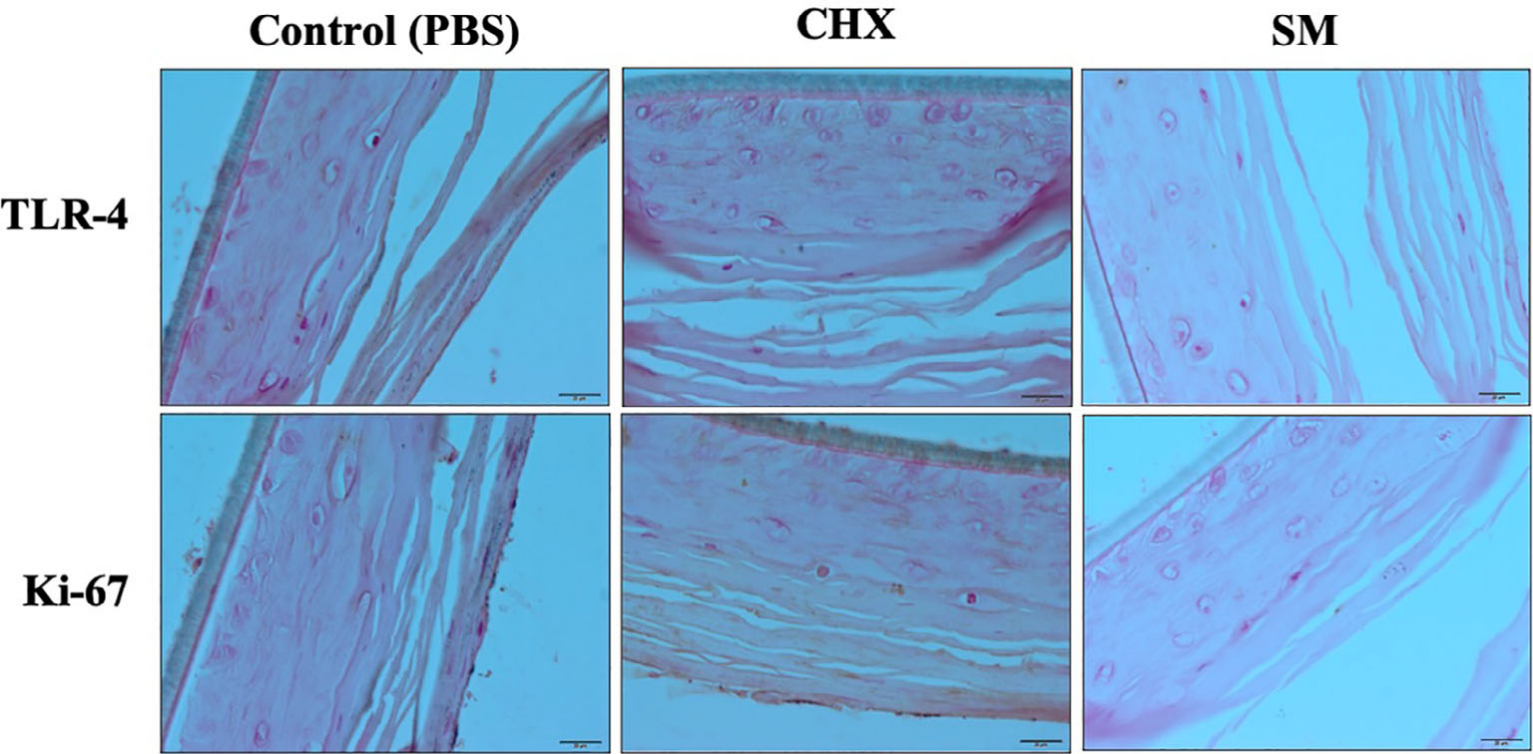
Immunohistochemistry images of LPS‐challenged Epi‐Gingival tissues showing TLR‐4 and Ki‐67 expression 24 h after respective treatment. Expression for TLR‐4 and Ki‐67 is represented by brown staining

TLR‐4, a pattern recognition receptor that recognizes *P. gingivalis* LPS (Kikkert et al., 2007), and Ki‐67, a cell proliferation marker and an indicator of the healing process, were both evaluated by immunohistochemistry in this study. Six hours after each treatment, TLR‐4 was expressed for all treatment groups in the lower layers of the tissue sections as indicated by the brown staining along the cell membranes (Figure 3). Twelve hours after initial treatments, TLR‐4 was highly expressed in all layers of PBS and CHX treated tissues, while in the SM treated tissues expression was minimal and only expressed in the keratinized layer and absent in the underlying cells (Figure 4). Twenty four hours after respective treatments, tissues treated with PBS showed high expression of TLR‐4 in all tissue layers (Figure 5). TLR‐4 was expressed slightly in both layers for CHX‐treated tissues and expression was negligible in SM treated tissues.

The proliferation marker, Ki‐67, was present in the superficial layer of PBS and SM treated tissues at 6 h post‐treatment, however, SM treatment group highly expressed Ki‐67 in the underlying layer. Ki‐67 was expressed minimally in the superficial layer of CHX treated tissues (Figure 3). At 12 h, Ki‐67 is expressed in the keratinized layer for all treatment groups. However, Ki‐67 is being expressed in the underlying cells after treatment with SM (Figure 4). And finally, at 24 h post‐treatment, Ki‐67 was highly expressed in all layers of CHX treated tissues, minimally expressed in the superficial layer of PBS treated tissues, and negligible in the SM treatment group (Figure 5).

## 4 DISCUSSION

Our data suggest that SM has anti‐inflammatory properties that may support its use as an alternative to CHX when adjunct therapy using mouth rinses is appropriate, especially in light of the clinical research indicating better patient compliance previously mentioned (Miley et al., 2019). Both SM and CHX appeared to reduce secretion of the potent pro‐inflammatory cytokine IL‐1β, a marker of active inflammation (Toker et al., 2008), IL‐1β's role in bone resorption and tissue destruction in relation to periodontitis has been described (Stashenko et al., 1987, 1991). Higher levels of this cytokine are found in both the saliva and gingival crevicular fluid of periodontitis patients (Kinney et al., 2014; Offenbacher et al., 2007; Rangbulla et al., 2017; Sánchez et al., 2013). While much of the IL‐1β in vivo originates with immune cells, gingival epithelial cells also secrete this cytokine and act as an important member of the innate immune defenses (Sandros et al., 2000). Furthermore, because of IL‐1β's prominence in periodontitis, it has been considered as a potential target for new therapeutics (Cheng et al., 2020). In this in vitro model of gingival epithelium, SM treatment, like treatment with CHX, resulted in a reduction of IL‐1β levels, supporting its use as a possible alternative to CHX as an adjunct therapy to reduce inflammation when appropriate.

IL‐8, a neutrophil chemoattractant and activator, has been used as a marker for inflammatory conditions (Shahzad et al., 2010), including periodontal disease (Finoti et al., 2017). IL‐8 recruits neutrophils to areas of inflammation where they are generally the first of the innate immune cells to arrive. In the periodontium, IL‐8 can be secreted by a variety of cells, including epithelial cells (Eckmann et al., 1993), in response to bacterial products such as LPS. Local levels of this chemokine have been shown to be higher in patients with chronic periodontitis compared to healthy controls (Finoti et al., 2017). At the 24‐h time point in this study, levels of IL‐8 were lower in the SM group compared to control, with CHX‐treated tissues showing intermediate levels of this chemokine.

TLR‐4 expression was also reduced by SM compared to PBS and CHX in our tissues. This was particularly evident at the 24‐hr time point. TLR‐4 not only responds to bacterial molecular patterns, but also to signals secreted by damaged cells (Molteni et al., 2016). Our tissues were exposed to both *P. gingivalis* LPS and to a wounding protocol. At the earliest time point, 6 h, TLR‐4 was expressed in all tissues. However, by 24 h, SM treated tissues had negligible TLR‐4 expression.

The cell proliferation marker active in all phases of the cell cycle, Ki‐67, was measured in wounded Epi‐Gingival tissues and was highly expressed in SM treated tissues. Furthermore, SM tissues expressed Ki‐67 earlier than CHX‐treated tissues. Zinc plays important roles in the regulation of DNA synthesis and stimulation of cell proliferation (MacDonald, 2000). In wound repair studies, it has been suggested that zinc may be important for cell membrane repair, cell migration, and extracellular matrix formulation (Lin et al., 2017). Other in vitro studies have suggested that resupply of zinc to quiescent zinc‐deficient cells can encourage restart of the cell cycle (Lo et al., 2020). SM is unique in that activated zinc ions are made available to oral tissues during its use. In our study, the available zinc ions may have supported the proliferation of the cells.

Overall, our in‐vitro data suggest that SM can be as or more effective than CHX mouth rinse in reducing inflammation, by reducing secretion of pro‐inflammatory cytokines, and expression of TLR‐4. Inflammation is a protective mechanism to help tissues such as those in the oral cavity deal with trauma or pathogenic invasion. However, once the inflammatory response has done its job, anti‐inflammatory responses must be allowed to restore order. Aberrant and uncontrolled inflammation is believed to be a significant driver in periodontal disease. While CHX treatment in this study reduced some inflammatory markers, SM treatment decreased these further. SM is not associated with the unpleasant side effects that are associated with CHX, such as tooth staining or taste alteration (Albert‐Kiszely et al., 2007). Furthermore, SM has a higher rate of patient compliance and was as effective as CHX for positive patient outcome in a clinical trial (Miley et al., 2019). Our work sets the stage for further study of SM as a viable alternative to CHX as an adjunct therapy for periodontal patients.

## CONFLICT OF INTEREST

The authors declare no conflicts of interest.

## AUTHOR CONTRIBUTIONS

Kaitlyn A. Stanton helped in the design and execution of the project, wrote the first draft of the manuscript, and contributed to the editing and completion of the final manuscript. Barbara A. McCracken helped in the design and execution of the project, data analysis, and writing of the manuscript.

## ETHICS STATEMENT

The project was reviewed by our internal research committee. No animals or human subjects were used in this study.

## Data Availability

The data that support the findings of this study are available from the corresponding author upon reasonable request.
